# Osa-miR162a fine-tunes rice resistance to *Magnaporthe oryzae* and Yield

**DOI:** 10.1186/s12284-020-00396-2

**Published:** 2020-06-10

**Authors:** Xu-Pu Li, Xiao-Chun Ma, He Wang, Yong Zhu, Xin-Xian Liu, Ting-Ting Li, Ya-Ping Zheng, Ji-Qun Zhao, Ji-Wei Zhang, Yan-Yan Huang, Mei Pu, Hui Feng, Jing Fan, Yan Li, Wen-Ming Wang

**Affiliations:** 1 State Key Laboratory of Crop Gene Exploration and Utilization in Southwest China, Sichuan Agricultural University, Chengdu, China; 2 Rice Research Institute and Key Lab for Major Crop Diseases, Sichuan Agricultural University, Chengdu, China

**Keywords:** Osa-miR162, *OsDCL1*, Blast disease resistance, Yield traits, Hydrogen peroxide, Defense-related gene

## Abstract

MicroRNAs (miRNAs) play essential roles in rice immunity against *Magnaporthe oryzae*, the causative agent of rice blast disease. Here we demonstrate that Osa-miR162a fine-tunes rice immunity against *M. oryzae* and yield traits. Overexpression of Osa-miR162a enhances rice resistance to *M. oryzae* accompanying enhanced induction of defense-related genes and accumulation of hydrogen peroxide (H_2_O_2_). In contrast, blocking Osa-miR162 by overexpressing a target mimic of Osa-miR162a enhances susceptibility to blast fungus associating with compromised induction of defense-related gene expression and H_2_O_2_ accumulation. Moreover, the transgenic lines overexpressing Osa-miR162a display decreased seed setting rate resulting in slight reduced yield per plant, whereas the transgenic lines blocking Osa-miR162 show an increased number of grains per panicle, resulting in increased yield per plant. Altered accumulation of Osa-miR162 had a limited impact on the expression of rice *Dicer-like 1* (*OsDCL1*) in these transgenic lines showing normal gross morphology, and silencing of *OsDCL1* led to enhanced resistance to blast fungus similar to that caused by overexpression of Osa-miR162a, suggesting the involvement of *OsDCL1* in Osa-miR162a-regulated resistance. Together, our results indicate that Osa-miR162a is involved in rice immunity against *M. oryzae* and fine-tunes resistance and yield.

## Background

Plants mount a multi-layered immune system in fighting against the invasion of pathogens, and small RNAs are essential regulators in this process (Katiyar-Agarwal and Jin 2010; Weiberg et al. 2014). miRNAs are a subset of non-coding small RNAs of 20–24 nucleotides (nt) and play vital roles in the regulation of gene expression either by chromatin methyl modification, mRNA cleavage, or translational inhibition (Yu et al. 2017). In plants, miRNAs are transcribed from miRNA genes and subsequently cleaved by the endoribonucleases DCL proteins, and processed into approximately 20–24 nt miRNAs (Kurihara and Watanabe 2004). Therefore, DCL1 plays important roles in the formation of mature miRNAs. In turn, the expression of *DCL1* is regulated by miR162 (Tijsterman and Plasterk 2004; Xie et al. 2004; Wu et al. 2009; Zhou et al. 2010).

miR162 is involved in multiple abiotic stress responses in plants. For example, in Arabidopsis, miR162 was induced by ABA treatment to improve adaptation to drought stress through suppressing *Trehalase precursor 1* (*OsTRE1*) (Tian et al. 2015). In maize, miR162 was responsive to salt stress with enhanced accumulation at 0.5 h post-treatment of salt, whereas decreased at 5 and 24 h post-treatment (Ding et al. 2009). In switchgrass (*Panicum virgatum*), the accumulation of miR162 was significantly changed under drought stress (Sun et al. 2012). In cotton (*Gossypium hirsutum L.*), miR162 was responsive to salinity (Salih et al. 2018). Besides, miR162 also participates in biotic stress responses. For example, virus suppressors, such as *Tombusvirus* p19 and *Cucumovirus* 2b, had a binding preference for miR162 to counteract host antiviral RNA silencing, a plant immune response during viral infection initiation (Pertermann et al. 2018).

In Arabidopsis and rice, miR162 suppresses the expression of *DCL1*, which plays essential roles in miRNA biogenesis and metabolism (Tijsterman and Plasterk 2004; Xie et al. 2004; Zhou et al. 2010). In Arabidopsis, the loss-of-function mutants of *AtDCL1* showed multiple altered phenotypes associating with the reduction of miRNA levels (Park et al. 2002). In rice, strong loss of function of *OsDCL1* led to developmental arrest at the seedling stage. In contrast, weak loss of function of *OsDCL1* resulted in pleiotropic developmental defects, including dark green, dwarfism, and different leaves and root phenotypes in comparison with control (Liu et al. 2005). Besides the roles in development, *DCL1* also regulates resistance against pathogens. For example, *AtDCL1* was required for miR393-mediated anti-bacterial defenses (Navarro et al. 2006). The silence of *OsDCL1* enhanced resistance to the virulent *Magnaporthe oryzae* strains with enhanced induction of defense responses, including hydrogen peroxide accumulation and cell death at the infected sites (Zhang et al. 2015). In contrast, activation of *OsDCL1a* enhances susceptibility to fungal pathogens *Fusarium fujikuroi* and *M. oryzae* accompanying with compromised pathogen-inducible defense responses, and impacts miRNA network (Salvador-Guirao et al. 2019). These results indicate that *OsDCL1* regulates rice growth and immunity against *M. oryzae*. Although Osa-miR162 suppresses the expression of Os*DCL1* (Zhou et al. 2010), the effect of Osa-miR162 on rice blast resistance and yield is unclear. It is also unknown whether there are other target genes of Osa-miR162 involved in these processes.

In rice, the Osa-miR162 family has two isoforms, namely Osa-miR162a and Osa-miR162b. Previously, we found that Osa-miR162a was responsive to *M. oryzae* in both susceptible and resistance accessions (Li et al. 2014). Here we showed that Osa-miR162 fine-tuned rice growth and blast disease resistance. Our data demonstrated that overexpression of Osa-miR162a led to enhanced resistance but penalized yield, whereas blocking Osa-miR162 via a target mimic resulted in suppressed resistance but increased yield. In addition, *OsDCL1* is involved in miR162-mediated resistance against *M. oryzae*.

## Results

### Osa-miR162a Is Differentially Responsive to *M. oryzae* in Susceptible and Resistant Accessions

**Fig. 1:**
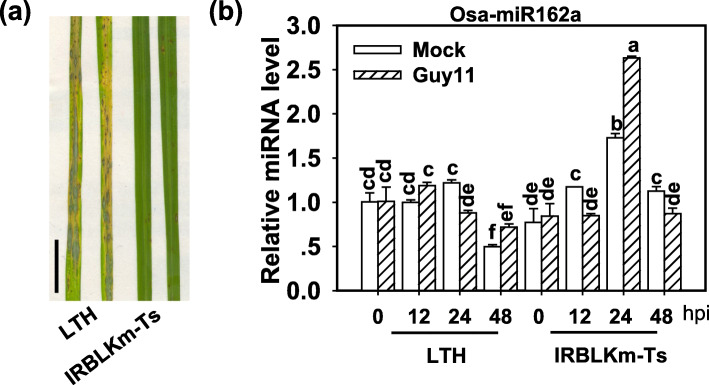
Osa-miR162a is differentially responsive to *Magnaporthe oryzae* in the susceptible and resistance accessions. **a** Blast disease phenotype on leaves of susceptible accession LTH and resistant accession IRBLKm-Ts following spray-inoculation of *Magnaporthe oryzae* Guy11 (1 × 10^5^ spore/ml concentration) at 5 days post-inoculation (dpi). Scale bar =1 cm. **b** Accumulation of Osa-miR162a in indicated accessions with or without Guy11 infection. Total RNA was used to carry out reverse-transcription (RT) with an Osa-miR162 specific stem-loop RT primers (Additional file 4: Table S1), and the RT product was subsequently used as a template for quantitative polymerase chain reaction (q-PCR) to detect the amounts of Osa-miR162. snRNA U6 served as an internal reference. Error bars indicate SD. Different letters above the bars indicate a significant difference (*P* < 0.05) as determined by a one-way ANOVA analysis. Similar results were obtained in at least two independent experiments

To explore the involvement of Osa-miR162a in rice blast resistance, we examined the amounts of Osa-miR162a in a highly susceptible accession Lijiang xin Tuan Heigu (LTH) and a resistant accession IRBLKm-Ts. LTH is sensitive to over 1300 regional isolates of *M. oryzae* worldwide and often used as a susceptible reference in blast disease assay (Lin et al. 2001). IRBLKm-Ts carries a resistance (*R*) locus *Pi-km* and exhibits resistance to *M. oryzae* isolates carrying *Avr-Pikm* (Tsumematsu et al. 2000). We identified the disease phenotype of LTH and IRBLkm-Ts by spray-inoculation of *M. oryzae* strain Guy11. LTH showed susceptible phenotype with serious disease lesions, whereas IRBLkm-Ts showed resistant phenotype with few small lesions (Fig. 1a). LTH displayed unchanged or decreased Osa-miR162a abundance following the inoculation of Guy11 in comparison with mock samples. In contrast, IRBLKm-Ts showed reduced accumulation at 12 and 48 hpi but increased at 24 hpi (Fig. 1b), indicating Osa-miR162a was involved in rice-*M. oryzae* interaction.

### Overexpression of Osa-miR162a Enhances Rice Resistance to *M. oryzae*

**Fig. 2:**
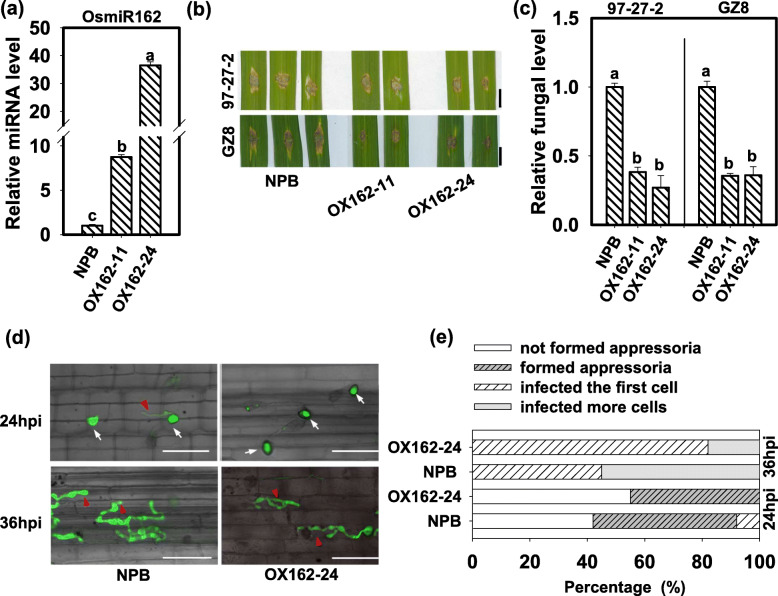
Overexpression of Osa-miR162a enhances rice resistance to *Magnaporthe oryzae*. **a** Accumulation of Osa-miR162a in transgenic lines harboring 35S: *Osa-miR162a* (OX162). Total RNA was used to carry out reverse-transcription with an Osa-miR162 specific stem-loop RT primers (Additional file 4: Table S1), and the RT product was subsequently used as a template for quantitative polymerase chain reaction (q-PCR) to detect the amounts of Osa-miR162. snRNA U6 served as an internal reference. **b** Blast disease phenotypes on leaves at 5 days post-inoculation of *M. oryzae* strain GZ8 and 97–27-2, respectively. Bar = 5 mm. **c** Relative fungal biomass of GZ8 and 97–27-2 on NPB and OX162. The relative fungal biomass was measured by using the ratio of DNA level of *M. oryzae Pot2* gene against the rice genomic ubiquitin DNA level. **d** Invasion process of *M. oryzae* strain GZ8 at 24 and 36 h post-inoculation (hpi) on sheath cells of indicated lines. Bars = 25 μm. The white arrows indicate appressoria formed from conidia, and the red arrowheads indicate invasive hypha in rice sheath cells. **e** Quantification analysis on infection process. Over 200 conidia in each line were analyzed. For (**a**) and (**c**), Error bars indicate SD (n = 3). Different letters above the bars indicate significant differences (*P* < 0.05) as determined by One-way ANOVA analysis. Similar results were obtained in at least two independent experiments

To explore the roles of Osa-miR162a in rice blast resistance, we constructed transgenic lines overexpressing Osa-miR162a (OX162) under Nipponbare (NPB) background. We got over 20 transgenic lines displaying increased amounts of mature Osa-miR162a in comparison with NPB control. We selected two lines showing moderate accumulation of Osa-miR162a and producing seeds, namely OX162–11 and OX162–24, for subsequent study (Fig. 2a). We then examined the blast disease phenotype of these lines following the punch-inoculation of two virulent strains, namely GZ8 and 97–27-2. GZ8 was an enhanced Green Fluorescence Protein (GFP)-tagged strain Zhong 8–10-14, which was derived from the paddy yard in China, and 97–27-2 was a strain derived from the paddy yard in Sichuan Province, China. OX162 lines displayed enhanced resistance with smaller disease lesions than control (Fig. 2b). Consistently, the fungal biomass in OX162 was significantly less than that in control (Fig. 2c). Then we examined the disease phenotype of OX162 by spray-inoculation of GZ8 and got the results similar to that by punch-inoculation (Additional file 1: Figure S1). Moreover, we also identified the resistance of OX162 against four *M. oryzae* strains isolated from paddy yard in Sichuan Province, China. Similarly, OX162 displayed smaller disease lesions than control (Additional file 2: Figure S2). These results indicate that overexpression of Osa-miR162a enhances rice blast resistance.

To understand how OX162 lines suppress fungal growth, we observed the infection process of GZ8 in leaf sheath. At 24 hpi, more than 55% of spores formed appressoria, and quite a few of which formed invasive hyphae in sheath cells of WT. However, in OX162, less than 50% of spores developed appressoria, and no invasive hyphae were observed (Fig. 2d). At 36 hpi, more than 50% of the spores started to infect the neighbor cells near the local infected cell of WT, whereas less than 20% of the spores invaded in the neighbor cells of OX162 (Fig. 2d). The quantification assay confirmed that overexpression of Osa-miR162a delayed the infection progress of blast fungus (Fig. 2e).

**Fig. 3:**
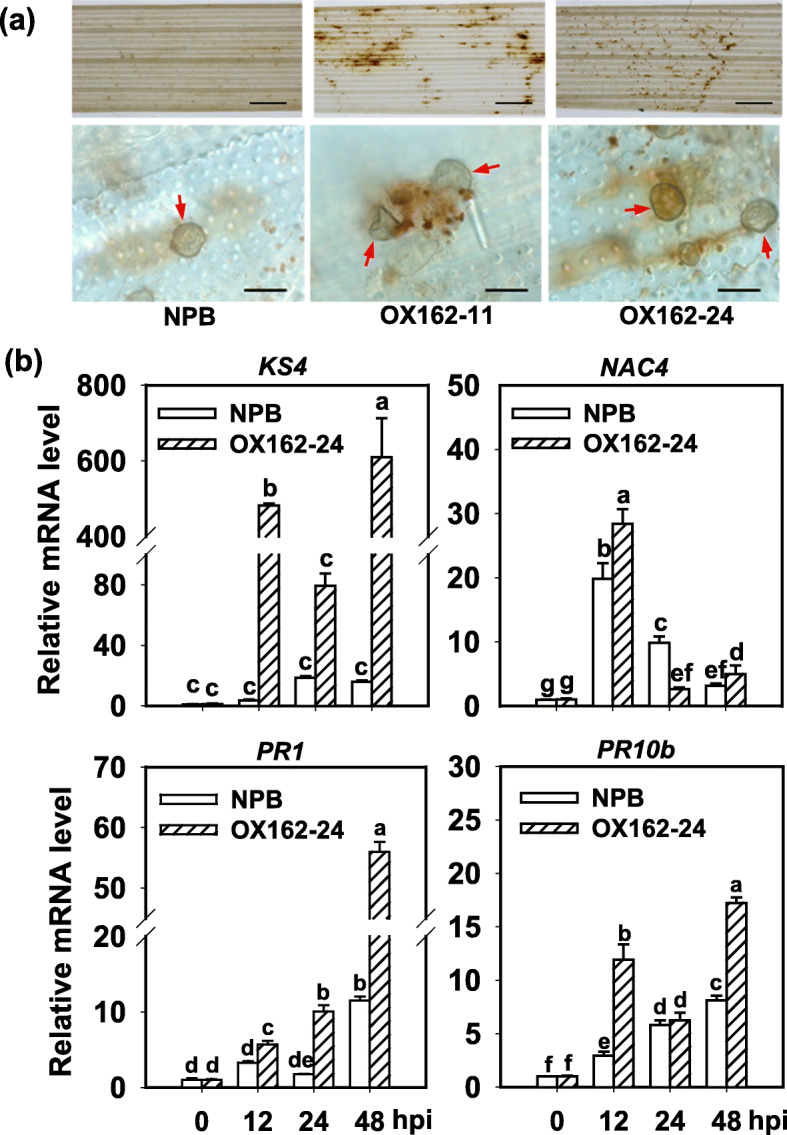
Osa-miR162a enhances the induction of disease-related defense responses. **a** Hydrogen peroxide (H_2_O_2_) accumulation in wild type (Nipponbare, NPB) and OX162 at 48 h post-inoculation (hpi) of *M. oryzae* strain GZ8. The intensity of brown indicates the amounts of H_2_O_2_, which was stained by 3,3′-diaminobenzidine (DAB). The red arrows indicate appressoria formed from conidia. The above photos were taken with a stereo-microscope, scale bars = 1 mm. The photos at the down portion were taken with a microscope (Zeiss imager A2), scale bars = 40 μm. **b** Relative mRNA levels of the defense-related genes (*OsKS4, OsNAC4, OsPR1*, and *OsPR10b*) in NPB and OX162 following inoculation of *M. oryzae* GZ8. Rice ubiquitin (*UBQ*) gene was served as an internal reference. The mRNA levels were normalized to that in NPB at 0 hpi. Error bars indicate SD (n = 3). Different letters above the bars indicate significant differences (*P* < 0.05) as determined by One-way ANOVA analysis. Similar results were obtained in at least two independent experiments

To understand why overexpression of Osa-miR162a delayed the infection of *M. oryzae*, we examined immune responses, such as H_2_O_2_ accumulation and induction of defense-related genes in OX162 and control. OX162 showed more H_2_O_2_ accumulation in the leaf cells around the infected sites than NPB following GZ8 inoculation (Fig. 3a). Four defense-related genes, namely *ENT-KA RENE SYNTHASE 4* (*KS4*), *NAC DOMAIN-CONTAINING PROTEIN 4* (*NAC4*), *PATHOGENESIS-RELATE GENG 1A* (*PR1a*), and *PR10b*, were examined in OX162 and NPB. *KS4* is an early-induced basal defense-related gene (Park et al. 2012). *NAC4* is involved in plant cell death and highly expressed in a lesion mimic mutant *spl11* at the lesion forming period (Yin et al. 2000). *PR1a* and *PR10b* are pathogenesis-related genes (Yamaguchi et al. 2013). The induction of *KS4* was enhanced to higher levels in OX162 than in NPB control at 12, 24 and 48 hpi, and *NAC4* was enhanced to higher levels in OX162 than in NPB control at 12 hpi and subsequently decreased at 24 hpi, then enhanced at 48 hpi again (Fig. 3b). Similarly, the expression of *PR1a* and *PR10b* was significantly induced to higher levels at 12 and 48 hpi than that in NPB control (Fig. 3b). These data indicate that overexpression of Osa-miR162a enhanced the immune responses triggered by *M. oryzae.*

### Blocking Osa-miR162a Leads to Increased Blast Disease Susceptibility

**Fig. 4:**
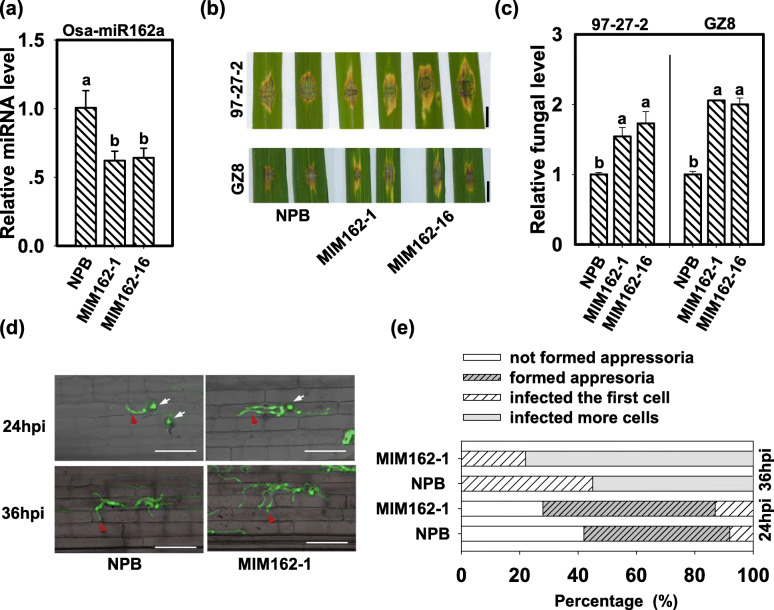
Expression of a target mimic of Osa-miR162a (MIM162) enhances rice susceptibility to *Magnaporthe oryzae*. **a** Accumulation of Osa-miR162a in wild type (Nipponbare, NPB) and MIM162. Total RNA was used for reverse-transcription (RT) with an Osa-miR162 specific stem-loop RT primers (Additional file 4: Table S1), and the RT product was subsequently used as a template for quantitative polymerase chain reaction (q-PCR). snRNA U6 served as an internal reference. **b** Blast disease phenotypes on leaves of NPB and MIM162 at 5 days post-inoculation (dpi) of *M. oryzae* strain GZ8 and 97–27-2. Bar = 5 mm. **c** Quantification analysis of fungal biomass in (**b**). The relative fungal biomass was determined by detecting the DNA levels of the *M. oryzae Pot2* gene against the rice *Ubiquitin* DNA levels. **d** Invasion process of GZ8 at 24 and 36 h post-inoculation (hpi) on sheath cells of the indicated lines. Bars = 25 μm. The white arrows indicate appressoria formed from conidia, and the red arrowheads indicate invasive hypha in rice sheath cells. **e** Quantification analysis on infection process. Over 200 conidia in each line were analyzed. For (**a**) and (**c**), Error bars indicate SD (n = 3). Different letters above the bars indicate significant differences (*P* < 0.05) as determined by One-way ANOVA analysis. Similar results were obtained in at least two independent experiments

To further confirm the roles of Osa-miR162a in rice immunity against blast fungus, we constructed the transgenic lines overexpressing a target mimic of Osa-miR162a (MIM162). MIM162 contained a sequence reversely complementary to Osa-miR162a with three nucleotides insertion between 10 to 11 nucleotide sites. Therefore, the target mimic acted as a sponge to absorb Osa-miR162 and block its binding to target genes (Additional file 3: Figure S3). We then examined the amounts of Osa-miR162a in MIM162. MIM162 displayed significantly less accumulation of Osa-miR162a than NPB control (Fig. 4a). Next, we conducted a disease assay on MIM162. As expected, MIM162 showed enhanced susceptibility to *M. oryzae* strain 97-27-2 and GZ8 with larger disease lesions following punch- or spray-inoculation (Fig. 4b and c, and Additional file 1: Figure S1). We also examined the disease phenotypes of MIM162 against four *M. oryzae* strains derived from the paddy yards by punch-inoculation. Similarly, MIM162 displayed larger disease lesions and supported more fungal growth than control (Additional file 2: Figure S2).

Moreover, the infection process of GZ8 in MIM162 was faster than that in control. At 24 hpi, more than 70% of spores formed appressoria or invasive hyphae in MIM162, in comparison with less than 60% of that in NPB control (Fig. 4d and e). At 36 hpi, more than 75% of spores invaded into the neighbor cells in MIM162, in comparison with less than 60% of that in NPB (Fig. 4d and e). These results indicate that blocking Osa-miR162 facilitates the invasion of *M. oryzae*.

**Fig. 5:**
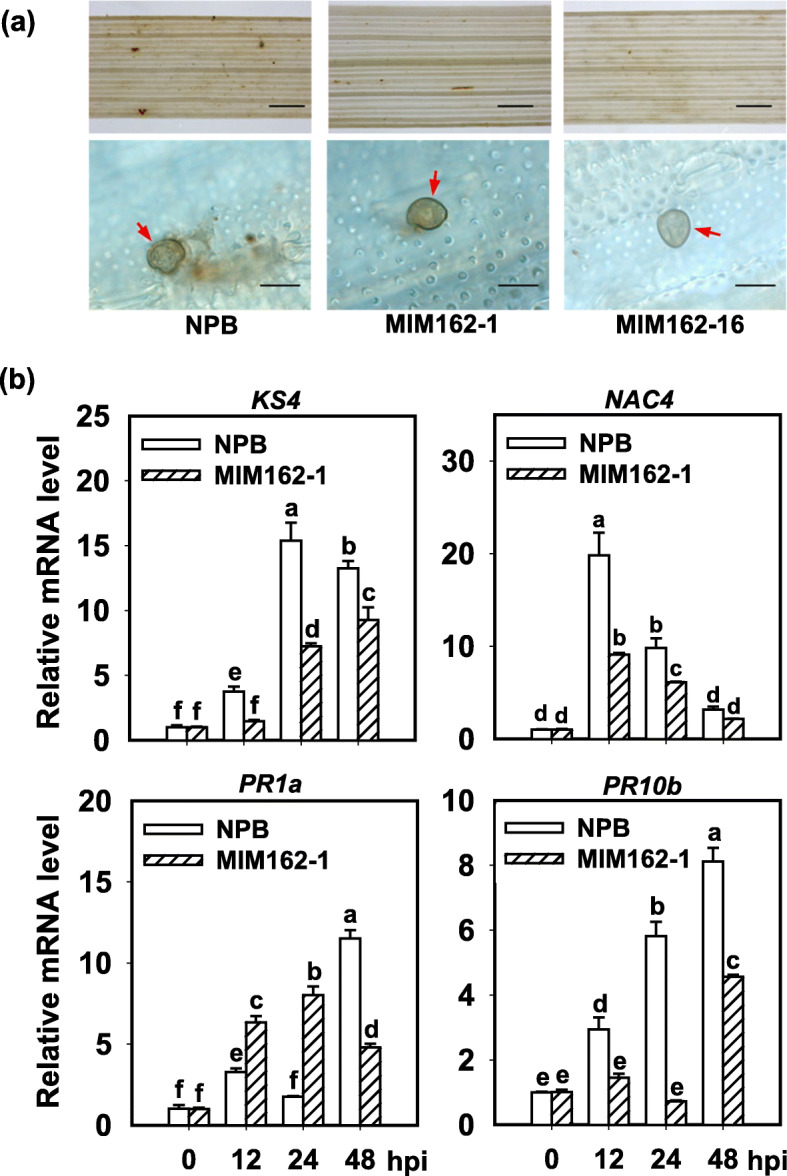
Blocking Osa-miR162a compromises the induction of disease-related defense responses. **a** Hydrogen peroxide (H_2_O_2_) accumulation in wild type (Nipponbare, NPB) and MIM162 at 48 h post-inoculation (hpi) of *M. oryzae* strain GZ8. The intensity of brown indicates the amounts of H_2_O_2_, which was stained by 3,3′-diaminobenzidine (DAB). The red arrows indicate appressoria formed from conidia. The upper photos were taken with a stereo-microscope, scale bars = 1 mm. The photos at the down portion were taken with a microscope (Zeiss imager A2), scale bars = 40 μm. **b** Expression of the defense-related genes (Os*KS4, OsNAC4, OsPR1,* and *OsPR10b*) in NPB and MIM162 following GZ8 inoculation. Rice ubiquitin (*UBQ*) gene was served as an internal reference. The mRNA levels were normalized to that in NPB at 0 hpi. Error bars indicate SD (n = 3). Different letters above the bars indicate significant differences (*P* < 0.05) as determined by One-way ANOVA analysis. Similar results were obtained in at least two independent experiments

We also examined the defense responses in MIM162. NPB plant showed visible H_2_O_2_ accumulation at the infected sites of leaves and cells following GZ8 infection, whereas MIM162 displayed little H_2_O_2_ amounts at the invasive sites (Fig. 5a). Consistently, the induction of defense-related genes, *KS4*, *NAC4*, *PR1a*, and *PR10b*, was lower in MIM162 than that in NPB (Fig. 5b). These data indicate that blocking Osa-miR162 compromises immune responses induced by *M. oryzae*.

### Osa-miR162a Regulates Rice Yield Traits

**Fig. 6:**
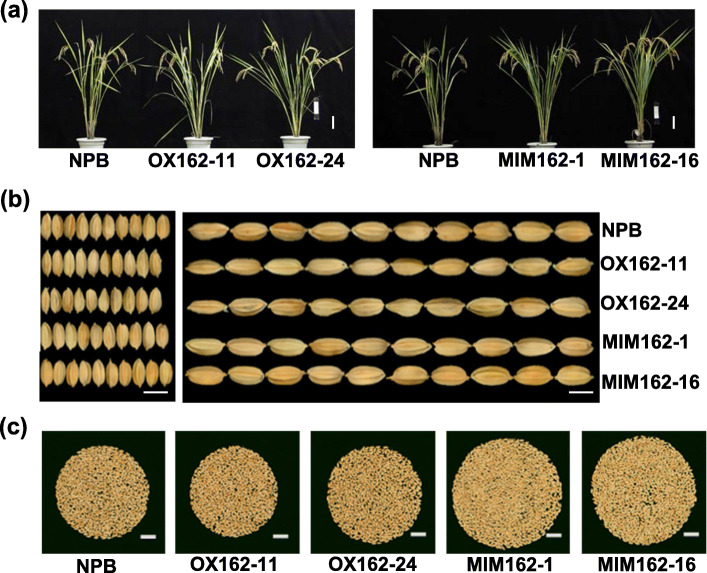
Osa-miR162a regulates rice yield. **a** Gross morphology of the wild type (Nipponbare, NPB), OX162, and MIM162 lines. Scale bars, 10 cm. **b** Photos of grains to show grain length and grain width of NPB, OX162, and MIM162 lines. Scale bars, 5 mm. **c** Photo of grains per plant of NPB, OX162, and MIM162 lines. Scale bars, 2 cm

We found that Osa-miR162a also controlled rice agronomic traits. Both OX162 and MIM162 exhibited comparable phenotypes with WT, including gross morphology, panicle number per plant, and panicle length (Fig. 6a and Table 1). However, OX162 showed narrower seeds, resulting in lower seed weight than control, and significantly lower seed setting rate, leading to lower grain number than control (Fig. 6b and Table 1). Substantially, OX162 displayed a slight lower yield per plant (Table 1). In contrast, MIM162 exhibited similar seed size as control (Fig. 6b), slightly lower seed weight (Table 1), lower seed setting rate, but more grains per plant in comparison with control (Fig. 6c and Table 1). As a result, MIM162 showed higher yield per plant than control (Table 1). These results indicate that Osa-miR162a compromises yield, whereas blocking Osa-miR162a enhances yield.

**Table 1:** The agronomic traits of OX162 and MIM162

Strain	Plant height (cm)	Panicle number	Panicle length (cm)	Setting Rate (%)	Grain number	1000-grain weight (g)	Yield per plant (g)
NPB	91.4 ± 2.4^a^	9.2 ± 0.44^a^	19.6 ± 0.74^a^	92.7 ± 0.93^a^	833 ± 45^a^	26.9 ± 0.63^a^	22.3 ± 2.35^ab^
OX-11	90.8 ± 2.16^a^	9.4 ± 1.34^a^	19.5 ± 0.63^a^	87.4 ± 1.52^a^	850 ± 74^a^	23.4 ± 0.56^c^	20.7 ± 3.90^b^
OX-24	89.6 ± 2.88^a^	10.6 ± 1.5^a^	19.6 ± 0.7^a^	60.7 ± 7.06^a^	839 ± 65^a^	22.8 ± 0.38^c^	18.3 ± 2.76^b^
MIM-1	94.8 ± 4.02^a^	9.4 ± 0.89^a^	19.8 ± 0.89^a^	90.5 ± 3.14^a^	987 ± 36^a^	25.9 ± 0.38^ab^	24.1 ± 3.35^a^
MIM-16	93.7 ± 4.4^a^	10.4 ± 0.89^a^	19.2 ± 0.94^a^	85.9 ± 0.77^a^	999 ± 71^a^	25.4 ± 0.3^b^	24.4 ± 3.75^a^

Notes: Data are shown as mean ± SD (n = 5). Means labeled with different letters indicate a significant difference at 5% level via Tukey-Kramer test

### *OsDCL1* Is Involved in Osa-miR162-Regulated Rice Blast Resistance

**Fig. 7:**
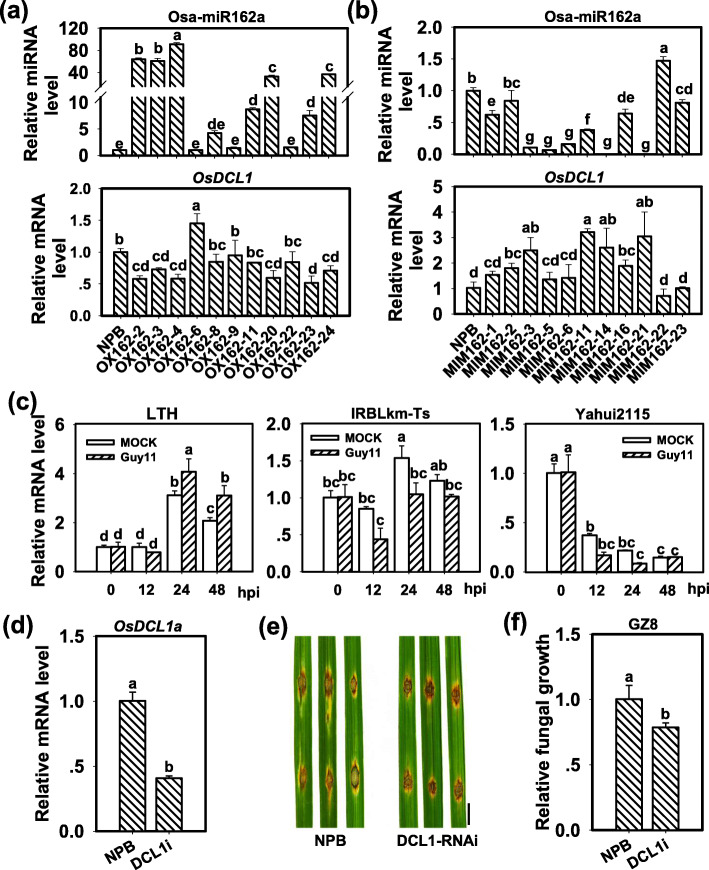
*OsDCL1* negatively regulates rice blast resistance. **a** and **b** Amounts of Osa-miR162a and mRNA levels of *OsDCL1* in OX162 (**a**) and MIM162 lines (**b**) in comparison with Nipponbare (NPB). Total RNA was used to carry out reverse-transcription (RT) with an Osa-miR162 specific stem-loop RT primer (Additional file 4: Table S1), and the RT product was subsequently used as a template for quantitative polymerase chain reaction (q-PCR) to detect the amounts of Osa-miR162. snRNA U6 served as an internal reference. **c** Expression of *OsDCL1* in susceptible accession LTH and resistance accessions (IRBLKm-Ts and Yahui2115) with or without *M. oryzae* strain Guy11 infection. RNA was extracted at the indicated time points for qRT-PCR analysis. The relative mRNA levels were normalized with the mRNA level of the mock sample at 0 hpi. **d** mRNA levels of *OsDCL1* in *OsDCL1* RNA inference transgenic line (DCL1i) and NPB plants. **e** Blast disease phenotypes on leaves of DCL1i and NPB at 5 days post-inoculation (dpi) of *M. oryzae* strain GZ8. Bar = 5 mm. **f** Quantification analysis of the fungal biomass in (**e**). Relative fungal biomass was measured by using the ratio of DNA level of *M. oryzae Pot2* gene against the rice genomic ubiquitin DNA level. For a, b, c, d, and f, error bars indicate SD (n = 3). Different letters above the bars indicate a significant difference (*P* < 0.05) as determined by a one-way ANOVA analysis. All the experiments were repeated two times with similar results

Rice Osa-miR162 was reported to target *LOC_Os03g02970* (Zhou et al. 2010) and was predicted to target *LOC_Os03g15230* (http://plantgrn.noble.org/psRNATarget/). *LOC_Os03g02970* encodes OsDCL1a, and *LOC_Os03g15230* encodes an expressed protein OsDUF292 (domains of unknown function protein 292; Additional file 3: Figure S3b). We examined the expression of Osa-miR162 and the two genes in 11 independent OX162 lines and 11 independent MIM162 lines, respectively. All the 22 detected lines displayed normal gross morphology and produced seeds. Seven of the 11 tested OX162 lines showed significantly higher Osa-miR162 accumulation (Fig. 7a). Conversely, six of the seven lines displayed significant decrease, and the one line showed slight decrease of *OsDCL1* mRNA levels (Fig. 7a). Besides, among the 11 tested MIM162 lines, nine lines displayed significantly lower Osa-miR162 abundance in comparison with NPB control (Fig. 7b). Conversely, five of the nine lines displayed significant increase of *OsDCL1* mRNA amounts, and three of the nine lines showed slight increase (Fig. 7b). These results indicated the regulation of Osa-miR162a on *OsDCL1*.

Intriguingly, the mRNA levels of *OsDCL1* decreased mildly in all the seven lines overexpressing Osa-miR162 (Fig. 7a). Even in the four OX162 lines showing over 40-fold amounts of Osa-miR162a, the mRNA levels of *OsDCL1* only decreased less than 50% of that of control (Fig. 7a), indicating that *OsDCL1* is critical for rice normal development, and Osa-miR162 had a limited impact on the expression of *OsDCL1* in these lines. However, the mRNA levels of *OsDUF292* were markedly higher in both OX162 and MIM162 (Additional file 3: Figure S3b), suggesting Osa-miR162 might not directly target *OsDUF292*.

To explore the roles of *OsDCL1* in rice resistance, we examined the expression of *OsDCL1* in susceptible accession LTH and resistance accessions IRBLKm-Ts and Yahui2115 upon *M. oryzae* infection, respectively. Yahui2115 is an elite hybrid restorer line carrying several blast resistance genes and widely used in breeding programs (Shi et al. 2015). The mRNA amounts of *OsDCL1* increased in LTH at 24 and 48 hpi of *M. oryzae* (Fig. 7c), and were reversely consistent with the amounts of Osa-miR162a in LTH following inoculation (Fig. 1b), indicating Os*DCL1* was involved in rice resistance to blast fungus and down-regulated by Osa-miR162a. In contrast, The mRNA amounts of *OsDCL1* were decreased in IRBLKm-Ts and Yahui2115 (Fig. 7c), further suggesting the involvement of *OsDCL1* in rice resistance to *M. oryzae*. We then examined the roles of *OsDCL1* in rice blast resistance by inoculation of GZ8 on an *OsDCL1* RNA inference line (DCL1i, (Liu et al. 2005)) showing significantly lower *OsDCL1* mRNA amounts than control (Fig. 7d). DCL1i line exhibited enhanced resistance to GZ8 with smaller disease lesions and supported less fungal growth (Fig. 7e and f), which was similar to the disease phenotype of OX162, and was consistent with the previous report that down-regulation of *OsDCL1* leads to enhanced resistance to blast fungus (Zhang et al. 2015). Together, these results indicate that *OsDCL1* possibly participates in Osa-miR162-regulated rice resistance against blast fungus.

## Discussion

MiRNAs play essential roles in controlling plant developmental progress and responses to biotic or abiotic stresses (de Lima et al. 2012; Seo et al. 2013). In recent years, rice-*M. oryzae* interaction has become a model in the study of plant-fungi interaction (Liu and Wang 2016). More than 70 miRNAs were responsive to *M. oryzae* or its elicitors and 11 of which have been identified as regulators in rice-*M. oryzae* interaction (Li et al. 2019b; Zhou et al. 2019). For example, seven miRNAs were identified as negative regulators in rice immunity against *M. oryzae* by disease assays on overexpressing or silencing transgenic lines, namely miR164a, miR169a, miR167d, miR319, miR396, miR444, and miR1873 (Li et al. 2017; Xiao et al. 2017; Chandran et al. 2018; Wang et al. 2018; Zhang et al. 2018a; Zhang et al. 2018b; Zhao et al. 2019; Zhou et al. 2019). In contrast, four miRNAs were characterized as positive regulators, namely miR160a, miR166k-miR166h, miR398b, and miR7695 (Campo et al. 2013; Li et al. 2014; Salvador-Guirao et al. 2018; Li et al. 2019a). Here we characterized Osa-miR162a as a positive regulator in rice immunity against *M. oryzae*. First, the expression of Osa-miR162a was unchanged or decreased in susceptible accession LTH but up-regulated in resistance accession IRBLKm-Ts following *M. oryzae* infection (Fig. 1). Second, overexpression of Osa-miR162a enhanced rice resistance with higher induction of defense responses (Figs. 2 and 3). In contrast, blocking Osa-miR162a by expressing a target mimic compromised rice resistance associating with the suppression of defense responses (Figs. 4 and 5).

Besides the regulation of blast disease resistance, Osa-miR162 also fine-tunes rice yield and yield traits. The trade-off between yield and disease resistance is a common phenomenon in crop production. For example, the mutation in *SQUAMOSA promoter-binding protein-like transcription factors 28* (*OsSPL28*) resulted in enhanced resistance to *M. oryzae* but led to a reduction in rice yield (Qiao et al. 2010). miRNAs may act as the crucial fine-tuning regulators to balance rice blast resistance and yield by regulating the expression of their target genes. For example, miR396 fine-tunes rice blast disease resistance and yield by controlling the expression of *Growth Regulating Factor* (*GRF*) transcription factor genes. The transgenic lines overexpressing miR396 showed enhanced susceptibility to *M. oryzae*, whereas the transgenic lines blocking miR396 exhibited enhanced rice resistance and increased yield (Chandran et al. 2018). In this study, we demonstrate that Osa-miR162a fine-tunes rice blast resistance and yield. Overexpression of Osa-miR162a leads to enhanced rice blast disease resistance but decreased yield, whereas blocking Osa-miR162 results in enhance susceptibility to *M. oryzae* but increased yield, indicating that the accurate accumulation of Osa-miR162a is necessary for the trade-off between yield and resistance.

Osa-miR162a possibly regulates rice-blast fungus interaction via *OsDCL1*-controlled miRNA network. *OsDCL1* RNAi lines displayed blast resistance similar to OX162: higher blast disease resistance (Fig. 7g-h) with increased induction of H_2_O_2_ and cell death at infected sites (Liu et al. 2005; Zhang et al. 2015). In the weak *OsDCL1*-knocking down mutant, more than ten miRNAs were identified as *OsDCL1*-regulated miRNAs for their differential expression in *OsDCL1* RNAi lines and control (Liu et al. 2005; Zhang et al. 2015). For example, the accumulation of eight miRNAs was abolished or significantly reduced in weak-loss-of-function transformants of *OsDCL1*, including miR156, miR159, miR166, miR167, miR168, miR168-3p, miR396, and miR528 (Liu et al. 2005). Besides, in another *OsDCL1* RNAi lines, the abundance of 12 miRNAs was decreased significantly, including miR156, miR166, miR167, miR168, and miR396 (Zhang et al. 2015). Intriguingly, among these miRNAs, four miRNAs were characterized as regulators in rice-*M. oryzae* interaction, namely miR166, miR167, miR169, and miR396 (Tang and Chu 2017), suggesting that Osa-miR162a possibly regulated rice-blast fungus interaction by controlling the miRNA network via *OsDCL1*.

Osa-miR162a possibly regulates rice yield traits via impact on *OsDCL1*, which is essential for rice normal development. *OsDCL1* RNAi lines with heavy loss of function showed developmental arrest at the seedling stage, including shoot and root abnormalities at an early developing stage, wilting of leaves and early senescence at a later stage, and eventually died without seed production (Liu et al. 2005), indicating OsDCL1 was essential for rice normal development. The lines with weak loss of function displayed pleiotropic developmental defects but with seed production (Liu et al. 2005). In this study, we found that OX162 lines with seed production all displayed a slightly impact on the expression of *OsDCL1*. In OX162 lines showing over 40-fold amounts of Osa-miR162a, the expression of *OsDCL1* decreased less than 50% of that of control (Fig. 7a), whereas, in MIM162 lines showing significant decreasing of Osa-miR162, the expression of *OsDCL1* was increased less than four-fold of that of control (Fig. 7b). We selected two OX162 lines showing an appropriate 10% to 20% decrease of *OsDCL1* amounts and two MIM162 lines displaying less than a 2-fold increase of *OsDCL1a* mRNA levels in comparison with that of control. The two OX162 lines showed slight defects in yield traits but enhanced blast disease resistance, suggesting that we could control the abundance of Osa-miR162a to manipulate resistance without significant loss of yield.

## Conclusions

Collectively, our results show that Osa-miR162a fine-tunes rice resistance and yield. Overexpression of Osa-miR162a leads to enhanced rice resistance to *M. oryzae* but slightly decreased yield per plant, whereas blocking Osa-miR162 results in enhanced susceptibility but increased yield per plant. Further study reveals that in the OX162 lines we studied, Osa-miR162 had a limited impact on the expression of *OsDCL1*, and silencing of which leads to similar resistance phenotype as that caused by overexpression of Osa-miR162a, indicating *OsDCL1* is involved in miR162-regulated resistance. Thus, Osa-miR162a positively regulates rice blast resistance and can be used to manipulate resistance without great penalty of yield.

## Materials and Methods

### Plant Materials and Growth Conditions

Rice plants used in this study included susceptible accession LTH, resistance accessions IRBLkm-Ts and Yahui2115. *Japonica* accession NPB was used to construct transgenic lines OX162 or MIM162a, respectively. The transgenic line silencing OsDCL1 is a generous gift from Prof. Xiaofeng Cao (Institute of Genetics and Developmental Biology, Chinese Academy of Sciences). For transformation, fungal inoculation, and defense responses assay, rice plants were planted at a growth room at 26 °C and 70% relative humidity with 14/10-h of day/night light. For yield traits detection, rice plants were planted in paddy yard at a regular season in Wenjiang district, Sichuan Province, China.

### Plasmid Construction and Genetic Transformation

The transgenic plants were generated following previous protocols (Li et al. 2017). To construct the transgenic lines overexpressing Osa-miR162*a*, the sequence of Osa-miR162a gene containing 400 bp upstream and 318 bp downstream sequences was amplified from NPB genomic DNA with primers Osa-miR162a-F and Osa-miR162a-R (Additional file 4: Table S1). Then the amplified fragment was cloned into *Kpn*I-*Sal*I sites of binary vector 35S-pCAMBIA1300 and got the overexpression construct p35S: *Osa-miR162a*. To construct the target mimic of Osa-miR162a, target mimic sequences of Osa-miR162a (CTGGATGCAGAAGTGGTTTATCGA) were formed by annealing with primers MIM162a-*BamH*I-F and MIM162a-*Bgl*II-R (Additional file 4: Table S1). The annealing double-strand product was inserted into *BamH*I- *Bgl*II sites of Arabidopsis *IPS1* gene to substitute the target site of miR399 as described previously (Franco-Zorrilla et al. 2007; Li et al. 2017). The reconstructed *IPS1-MIM162* sequence was cloned into *Kpn*I-*Sal*I sites of the binary vector pCAMBIA1300, resulting in overexpressing construct p35S: *MIM162*. The vectors were transformed into NPB by *Agrobacterium* strain EHA105, and the positive transgenic lines were screened with Hygromycin B.

### Pathogen Infection and Microscopy Analysis

*M. oryzae* strains Guy11, 97–27-2, GZ8, DZ-6, DZ-31, DZ-46, and DZ-110 were used in this study. 97–27-2 was a strain derived from paddy yard in Sichuan province, China, and GZ8 was a strain derived from a rice field in the north of China tagged with enhanced GFP. DZ-6, DZ-31, DZ-46, and DZ-110 were strains collected from the paddy yard in Dazhu county, Sichuan Province, China. The isolates were cultured in oatmeal/tomato media at 28 °C with 12-h/12-h light/dark cycles. Two weeks later, the hyphae were scratched, and the plates were continuously cultured at 28 °C with 24-h light for sporulation. After 4 days post-inoculation (dpi), spores were collected, and the inoculum concentration was adjusted to 5 × 10^5^ spore /ml for punch- and spray-inoculation. T3 seedlings of transgenic plants at three-leaf-stage were used for spray inoculation following previous reports (Qu et al. 2006). The disease phenotypes on the leaf two were recorded at 5 dpi. For punch-inoculation, the leaves of T3 seedlings at three-leaf-stage were slightly wounded with a mouse ear punch, and 5 μl of spore suspension was drop-inoculated on the wound. The disease phenotypes were recorded at 5 dpi, and the inoculated leaves were collected for fungal biomass assay. The relative fungal biomass was measured using the DNA amounts of fungal *Mopot2* against rice ubiquitin DNA amounts by quantitative PCR (Li et al. 2017). For invasive observation, the 5-cm-long leaf sheaths of the seedlings at five-leaf-stage were inoculated with spores suspension of eGFP-tagged *M. oryzae* strain GZ8 (2 × 10^5^ /ml) as described previously (Kankanala et al. 2007). The epidermal layer was excised for observation at 24 and 36 h post-inoculation (hpi). The fungal growth process was analyzed by Laser Scanning Confocal Microscopy (Nikon A1), and the infestation stage was analyzed as described previously (Li et al. 2014). For H_2_O_2_ detection, three-leaf-stage seedlings were spray inoculated with GZ8 (5 × 10^5^ /ml). The leaves were collected at 36 hpi and placed in 3, 3′-Diaminobenzidine (DAB) (1 mg /ml, Sigma, ALORICH, USA) for dying. The dying leaves were shook at room temperature for 12 h in a dark place, and then decolorized in 90% ethyl alcohol at 65 °C for several times (Xiao et al. 2003). The photo showing H_2_O_2_ accumulation on leaves was captured with a stereomicroscope (Zeiss imager). H_2_O_2_ accumulation in leaf cells and fungal structures were observed with a microscope (Zeiss imager A2).

### RNA Extraction and Quantitative Reverse-Transcription Polymerase Chain Reaction (qRT-PCR) Analysis

Three-leaf-stage plants were inoculated with or without *M. oryzae* by spraying spore suspensions at a concentration of 5 × 10^5^ /ml, and the leaves were collected at 0, 12, 24, and 48 hpi. Total RNA was extracted from these leaves using Trizol reagent (Invitrogen) and was used to detect the amounts of Osa-miR162 and gene expression. The qRT-PCR analysis was used to detect the amounts of Osa-miR162a and expression of genes. The RNA quality and quantity were determined by a spectrophotometer (NanoDrop2000 Uvevis). For analysis of amounts of Osa-miR162, Stem-loop pulse reverse-transcription was performed using the qPCR-RT Master Mix with gDNA Remover (TOYOBO) with Osa-miR162a stem-loop primer (Additional file 4: Table S1) following the direction. Then the RT products were used as templates for qPCR with the primers Osa-miR162a-RT-F and universal reverse primer (Additional file 4: Table S1) to detect the amounts of Osa-miR162 (Chen et al. 2005; Varkonyi-Gasic et al. 2007). For analysis of the expression of genes, the first-strand cDNA was synthesized using the qPCR-RT Master Mix with gDNA Remover (TOYOBO) following the direction. Then the expression of genes was detected with specific forward and reverse primers listed in Additional file 4: Table S1. U6 snRNA was used as an internal reference to normalize Osa-miR162 levels (Turner et al. 2013), and the rice ubiquitin (*UBQ*) gene was selected as an internal reference of the detected genes. qRT-PCR was performed using SYBR Green mix (Quanti Nova SYBR Green PCR Kit, QIGEN, Chengdu, China) with BIO-RAD C1000TM Thermal Cycler (Bio-Rad Inc., Chengdu, China).

### Yield Trait Measurements

NPB, OX162, and MIM162 lines were planted in paddy yard at a regular season in 2019 at Wenjiang District, Sichuan Province, China. Plant height, panicle number per plant, panicle length, seed setting rate, grain number per panicle, 1000-grain weight, and yield per plant were measured at full maturity from five plants. The filled grains were dried in a 42 °C oven for 1 week. The 1000-grain weight was measured using an SC-A grain analysis system (Wanshen Ltd., Hangzhou, China). All the data of yield traits were analyzed by One-Way ANOVA followed by post hoc Tukey HSD analysis at a significant level of 0.05.

## Supplementary information

**Additional file 1: Figure S1.** Overexpression of Osa-miR162a leads to enhanced resistance to *Magnaporthe oryzae* upon spray-inoculation. (a) Disease phenotypes on leaves of the indicated lines following spray-inoculation with *M. oryzae* strain GZ8. The phenotype was captured at 5 days post-inoculation. Bar = 5 mm. (b) Quantification analysis of the fungal biomass in (a). Relative fungal biomass was measured by using the ratio of DNA level of *M. oryzae Pot2* gene against the rice genomic ubiquitin DNA level. Error bars indicate SD (n = 3). Different letters above the bars indicate a significant difference (*P* < 0.05) as determined by a one-way ANOVA analysis. Similar results were obtained in at least two independent experiments.

**Additional file 2: Figure S2.** Overexpression of Osa-miR162a results in enhanced resistance to multiple *Magnaporthe oryzae* strains. (a) Disease phenotypes on leaves of NPB, OX162 and MIM162 following punch-inoculation with field-derived *M. oryzae* strains. The phenotype was captured at 5 days post-inoculation. Bars = 5 mm. (b-e) Quantification analysis of the fungal biomass in (a). Relative fungal biomass was measured by using the ratio of DNA level of *M. oryzae Pot2* gene against the rice genomic ubiquitin DNA level. Error bars indicate SD (n = 3). Different letters above the bars indicate a significant difference (*P* < 0.05) as determined by a one-way ANOVA analysis. Similar results were obtained in at least two independent experiments

**Additional file 3: Figure S3.** Osa-miR162a does not repress the expression of *DUF292*. (a) The alignment of Osa-miR162a and Osa-miR162b with MIM162. (b) The sequence alignment of Osa-miR162a with target sites of candidate genes. Mismatched nucleotides were highlighted in red colors. (c and d) Quantitative reverse transcription polymerase chain reaction (RT-qPCR) data indicate amounts of Osa-miR162a levels in OX162 (c) and MIM162 lines (d) in comparison with Nipponbare (NPB). For qPCR, Data are shown as mean ± SD (n = 3). Different letters indicate a significant difference at 5% level via Tukey-Kramer test. All the experiments were repeated two times with similar results.

**Additional file 4: Table S1.** Primers used in this study.

## Data Availability

The data sets supporting the conclusions of this article are included within the article and its additional files.

## Abbreviations

*M. oryzae*: *Magnaporthe oryzae*
miRNAs: MicroRNAs
*OsDCL1*: *Dicer-like 1*
H_2_O_2_: Hydrogen peroxide
nt: Nucleotides
AGO: ARGONAUTE
miRISC: MiRNA-Induced Silence Complex
*OsTRE1*: Trehalase precursor 1
LTH: Lijiangxin Tuan Heigu
*R*: Resistance
OX162: Transgenic lines overexpressing Osa-miR162a
GZ8: GFP-tagged Zhong 8–10-14
*KS4*: *ENT-KA RENE SYNTHASE 4*
*NAC4*: *NAC DOMAIN-CONTAINING PROTEIN 4*
*PR1a*: *PATHOGENESIS-RELATE GENG 1A*
MIM162: The transgenic lines overexpressing a target mimic of Osa-miR162a
OsDUF292: Domains of unknown function protein
DCL1i: *OsDCL1* RNA inference line
*OsSPL28*: SQUAMOSA promoter-binding protein-like transcription factors 28
*GRF*: *Growth Regulating Factor*
