# Supplementary figures and images for: Osa-miR162a fine-tunes rice resistance to Magnaporthe oryzae and Yield
Source: Rice (N Y). 2020 Jun 10;13:38. doi: 10.1186/s12284-020-00396-2 (PMC7287001; doi:10.1186/s12284-020-00396-2)

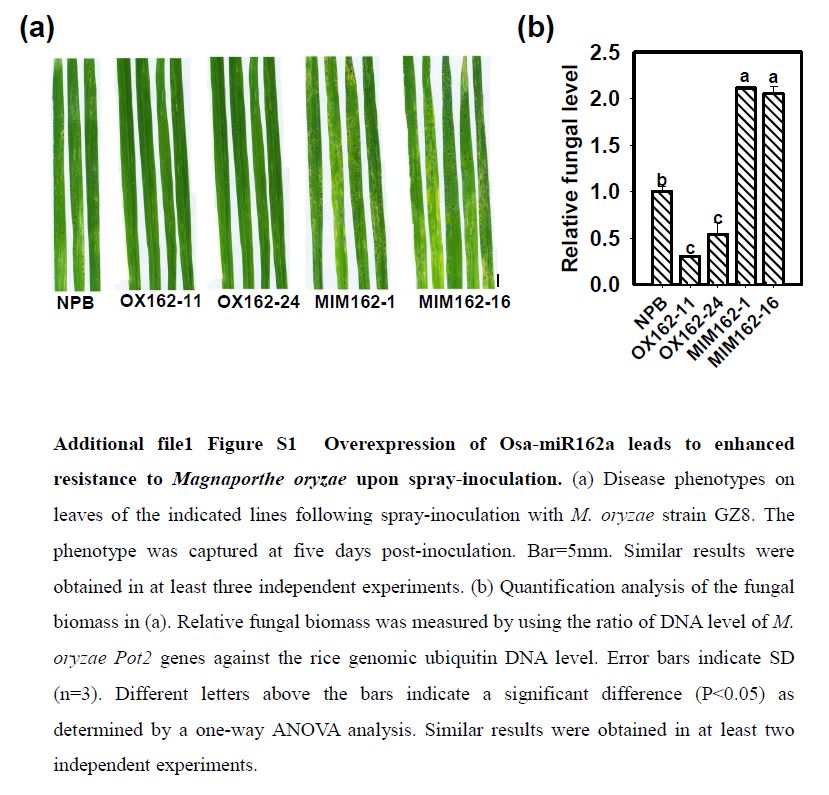

Supplement: Supplementary file 1 — Additional file 1: Figure S1. Overexpression of Osa-miR162a leads to enhanced resistance to Magnaporthe oryzae upon spray-inoculation. (a) Disease phenotypes on leaves of the indicated lines following spray-inoculation with M. oryzae strain GZ8. The phenotype was captured at 5 days post-inoculation. Bar = 5 mm. (b) Quantification analysis of the fungal biomass in (a). Relative fungal biomass was measured by using the ratio of DNA level of M. oryzae Pot2 gene against the rice genomic ubiquitin DNA level. Error bars indicate SD (n = 3). Different letters above the bars indicate a significant difference (P < 0.05) as determined by a one-way ANOVA analysis. Similar results were obtained in at least two independent experiments. [file 12284_2020_396_MOESM1_ESM.docx]

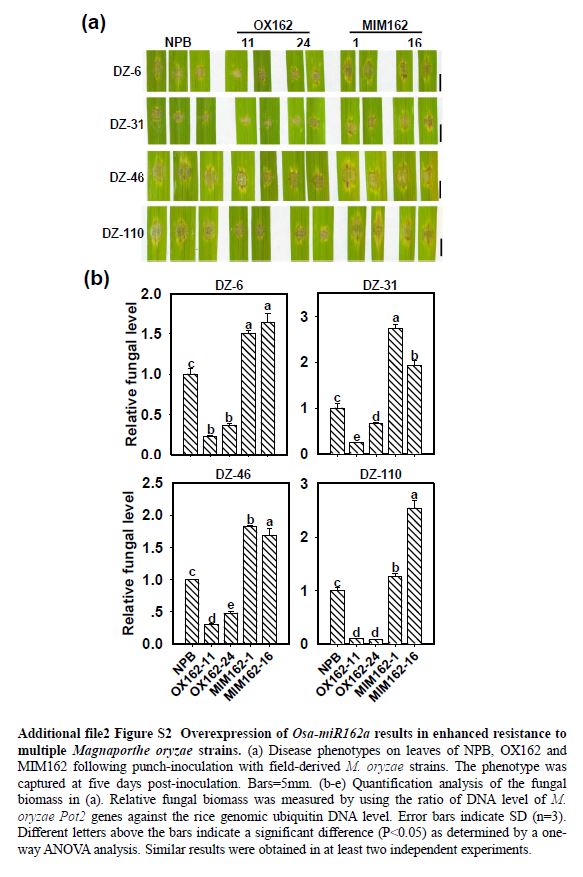

Supplement: Supplementary file 2 — Additional file 2: Figure S2. Overexpression of Osa-miR162a results in enhanced resistance to multiple Magnaporthe oryzae strains. (a) Disease phenotypes on leaves of NPB, OX162 and MIM162 following punch-inoculation with field-derived M. oryzae strains. The phenotype was captured at 5 days post-inoculation. Bars = 5 mm. (b-e) Quantification analysis of the fungal biomass in (a). Relative fungal biomass was measured by using the ratio of DNA level of M. oryzae Pot2 gene against the rice genomic ubiquitin DNA level. Error bars indicate SD (n = 3). Different letters above the bars indicate a significant difference (P < 0.05) as determined by a one-way ANOVA analysis. Similar results were obtained in at least two independent experiments [file 12284_2020_396_MOESM2_ESM.docx]

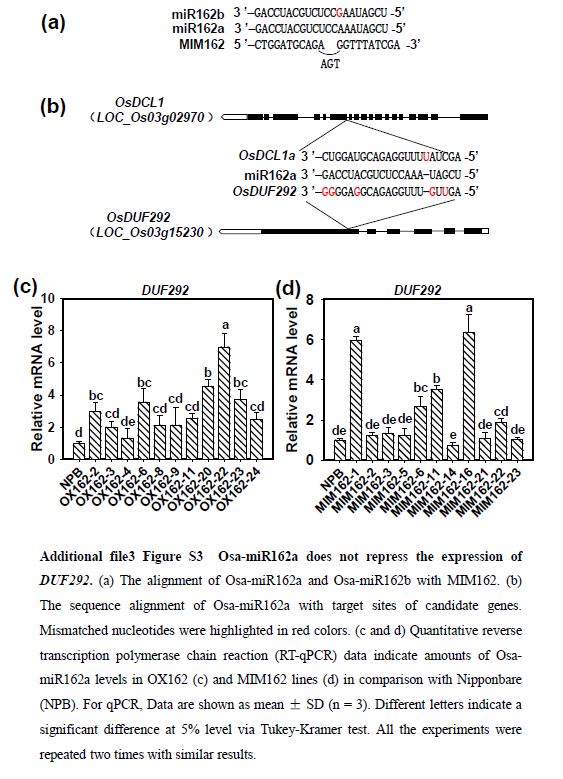

Supplement: Supplementary file 3 — Additional file 3: Figure S3. Osa-miR162a does not repress the expression of DUF292. (a) The alignment of Osa-miR162a and Osa-miR162b with MIM162. (b) The sequence alignment of Osa-miR162a with target sites of candidate genes. Mismatched nucleotides were highlighted in red colors. (c and d) Quantitative reverse transcription polymerase chain reaction (RT-qPCR) data indicate amounts of Osa-miR162a levels in OX162 (c) and MIM162 lines (d) in comparison with Nipponbare (NPB). For qPCR, Data are shown as mean ± SD (n = 3). Different letters indicate a significant difference at 5% level via Tukey-Kramer test. All the experiments were repeated two times with similar results. [file 12284_2020_396_MOESM3_ESM.docx]
