# Supplementary material for: Osa-miR162a fine-tunes rice resistance to Magnaporthe oryzae and Yield
Source: Rice (N Y). 2020 Jun 10;13:38. doi: 10.1186/s12284-020-00396-2 (PMC7287001; doi:10.1186/s12284-020-00396-2)
Supplement: Supplementary file 4 — Additional file 4: Table S1. Primers used in this study. [file 12284_2020_396_MOESM4_ESM.docx]

**Additional file 4 Table S1. Primers used in the study**

| Primers | Sequence(5'-3') | objective |
| --- | --- | --- |
| Osa-miR162a-F-kpn1 | ACAGGTACCACTACTACGTCGTCACTATAG | Make OX162a |
| Osa-miR162a-R-Sal1 | ACAGTCGACTATGGCTGATGCTAAGTCTC | Make OX162a |
| MIM162a-BamH1-F | TTCTGGATGCAGAGACGGTTTATCGAAGCTTCGGTTCCCCTCGGAATCA | Make MIM162 |
| MIM162a-BglII-R | CTTCGATAAACCGTCTCTGCATCCAGTAATTTCTAGAGGGAGATAAACA | Make MIM162 |
| Osa-miR162a RT-F | TCGATAAACCTCTGCATCCAG | qRT-PCR |
| universal reverse primer | CAGTGCAGGGTCCGAGGTAT | qRT-PCR |
| Osa-miR162a-stem loop | GTTGGCTCTGGTGCAGGGTCCGAGGTATTCGCACCAGAGCCAACCTGGAT | qRT-PCR |
| Osa-miR162LOC_Os03g15230-F | GCAGAAGCAGAGGCTGACTA | qRT-PCR |
| Osa-miR162LOC_Os03g15230-R | TGCTTGCCCTGATCGGTATC | qRT-PCR |
| Osa-miR162LOC_Os03g02970-F | CACAAGTGCATTCCTCGCTG | qRT-PCR |
| Osa-miR162LOC_Os03g02970-R | TACAAAGCATCCCGAGCAGG | qRT-PCR |
| OsNAC4-F for RT | TCCTGCCACCATTCTGAGATG | qRT-PCR |
| OsNAC4-R for RT | TTGCAGAATCATGCTTGCCAG | qRT-PCR |
| OsKS4-F for RT | TCGCATTGCGTGTGCAA | qRT-PCR |
| OsKS4-R for RT | TTGGAACTTCCGACATCGAAA | qRT-PCR |
| OsPR1a-F for RT | GGAAGTACGGCGAGAACATC | qRT-PCR |
| OsPR1a-R for RT | TGGTCGTACCACTGCTTCTC | qRT-PCR |
| OsPR10b-F for RT: | AACACGTGTGGTGGCACGTG | qRT-PCR |
| OsPR10b-R for RT: | TCATCTTGAGCATGCCGAAG | qRT-PCR |
| U6 RT-F | CGATAAAATTGGAACGATACAGA | qRT-PCR |
| U6 RT-R | ATTTGGACCATTTCTCGATTTGT | qRT-PCR |
| UBQ-F for RT | ACCCTGGCTGACTACAACATC | qRT-PCR |
| UBQ-R for RT | AGTTGACAGCCCTAGGGTG | qRT-PCR |
| MoPot2_F | ACGACCCGTCTTTACTTATTTGG | M. oryzea quantity identification gene |
| MoPot2_R | AAGTAGCGTTGGTTTTGTTGGAT |  |
